# Complete Genome Sequence of Vibrio coralliilyticus OCN008

**DOI:** 10.1128/MRA.00323-20

**Published:** 2020-07-23

**Authors:** Victoria N. Lydick, Douglas B. Rusch, Blake Ushijima, Julia C. van Kessel

**Affiliations:** a Biology Department, Indiana University, Bloomington, Indiana, USA; b Smithsonian Marine Station, Fort Pierce, Florida, USA; University of Delaware

## Abstract

Here, we report the complete genome sequence of Vibrio coralliilyticus OCN008, a marine bacterium that infects reef-building coral. Previous sequencing efforts yielded an incomplete sequence (210 contigs). We used Nanopore and Illumina sequencing data to obtain complete sequences of the two circular chromosomes (3.48 and 1.91 Mb) and one megaplasmid (244.69 kb).

## ANNOUNCEMENT

Vibrio coralliilyticus is a marine bacterium that infects multiple genera of coral and marine shellfish species (1–3). Recent experiments with the OCN008 strain have investigated its basic physiology, pathogenesis, and responses to changes in environmental signals (3–7). Strain OCN008 was isolated from the coral Porites compressa and subsequently discovered to infect Montipora capitata from the fringing reef of Moku o Lo‘e in Kāne‘ohe Bay, HI; its cultivation methods have been previously reported (5, 6). A draft genome sequence of 210 contigs was published and is available in GenBank (accession number AVOO00000000.1) (5). To complete a new, whole sequence of this genome and enable genetic and transcriptomic data analyses of OCN008, we resequenced the genome and *de novo* assembled the genome using new sequencing data.

Genomic DNA was extracted using the GeneJET genomic DNA purification kit (Thermo Scientific). The DNA integrity was analyzed using a 4200 TapeStation system (Agilent Technologies) and quantified using the Qubit double-stranded DNA (dsDNA) high-sensitivity (HS) assay kit (catalog number Q32854; Invitrogen). The Illumina Nextera DNA sequencing (DNA-seq) library was prepared with a Nextera DNA Flex library prep kit (Illumina); the standard manufacturing protocol was followed with one-fifth the standard reaction volume for all of the steps. The final Nextera DNA Flex library was analyzed with the Agilent Technologies 4200 TapeStation system. Sequencing was performed using an Illumina NextSeq 500/550 system (v2 kits) with a 75-bp sequencing module, generating 42-bp paired-end reads. Additionally, a Nanopore genomic DNA-seq library was prepared using a Nanopore genomic DNA by ligation kit (catalog number SQK-LSK109). The final library was quantified by using a Qubit dsDNA HS assay kit, and sequencing was performed using a MinION R9.4.1 flow cell (catalog number FLO-MIN106).

The Nanopore data were basecalled using the graphics processing unit (GPU)-enabled Guppy algorithm (version 3.4.4+a296acb) (8). The primary assembly was performed with Canu (version 1.8) (9) with the following parameters: genomeSize, 5m; maxThreads, 20; and -nanopore-raw combined.fastq. Illumina reads (6,127,597 read pairs) were trimmed with Trimmomatic (version 0.38) (10) with the following parameters: ILLUMINACLIP:adapters.fa:3:20:6, LEADING:3, TRAILING:3, SLIDINGWINDOW:4:20, and MINLEN:40. Only 2.53% of the reads were dropped. The quality of the reference was tested and corrected using breseq (version 0.33.2) (11). The breseq output was used with custom Perl scripts to iteratively correct the reference (custom scripts are available on Github at https://github.com/Juliacvk/OCN008_ASM). After 6 iterations, the consensus converged and no longer had errors. The overall contiguity was verified by examining the alignment of the Nanopore reads to the corrected reference (minimap2 version 2.17-r943-dirty) (12). The final genome assembly was annotated using the NCBI Prokaryotic Genome Annotation Pipeline (PGAP) (13, 14).

The resulting genome assembly consists of two chromosomes and one megaplasmid. The assembly is full length with 3 contigs and a total size of 5.63 Mb (chromosome I [Chr I], 3.48 Mb; Chr II, 1.91 Mb; plasmid, 244.69 kb). The OCN008 genome has a G+C content of 45.7%, and those of individual molecules are 45.7% (Chr I), 45.3% (Chr II), and 49.7% (plasmid). The closest relative using RefSeq genomes compared with chromosome I of Vibrio coralliilyticus OCN008 in GenBank is Vibrio coralliilyticus SNUTY-1 chromosome I (97.5% nucleotide identity). The NCBI annotation revealed 4,989 protein-coding sequences, 12 5S rRNAs, 11 16S RNAs, 11 23S rRNAs, and 116 tRNAs. The contributions of this complete genome sequence assembly encourage further research into the functional roles of quorum response and virulence interaction among various vibrio and host species.

### Data availability.

The complete genome sequence was deposited in GenBank under the accession numbers CP048693, CP048694, and CP048695. Versions CP048693.1, CP048694.1, and CP048695.1 are described in this paper. The raw reads are available under BioProject number PRJNA605822 and Sequence Read Archive (SRA) Run Selector study accession number SRP262515.
